# A Small-Molecule Inhibitor of the Anthranilyl-CoA Synthetase PqsA for the Treatment of Multidrug-Resistant Pseudomonas aeruginosa

**DOI:** 10.1128/spectrum.02764-21

**Published:** 2022-07-20

**Authors:** Jianwei Chen, Yaojia Lu, Fei Ye, Hongfang Zhang, Yonglie Zhou, Jiangtao Li, Qiang Wu, Xuewei Xu, Qihao Wu, Bin Wei, Huawei Zhang, Hong Wang

**Affiliations:** a College of Pharmaceutical Science & Collaborative Innovation Center of Yangtze River Delta Region Green Pharmaceuticals, Zhejiang University of Technology, Hangzhou, China; b College of Life Sciences, Zhejiang Sci-Tech University, Hangzhou, China; c Zhejiang Provincial People’s Hospital, Hangzhou, China; d The Second Affiliated Hospital of Zhejiang University School of Medicine, Hangzhou, China; e State Key Laboratory of Quality Research in Chinese Medicines, Macau University of Science and Technology, Macau, China; f Second Institute of Oceanography, MNR, Hangzhou, China; Emory University School of Medicine

**Keywords:** PqsA, *P. aeruginosa*, quorum sensing, quorum sensing inhibitor

## Abstract

One of the challenges associated with the treatment of Pseudomonas aeruginosa infections is the high prevalence of multidrug resistance (MDR). Since conventional antibiotics are ineffective at treating such bacterial infections, innovative antibiotics acting upon novel targets or via mechanisms are urgently required. In this study, we identified a quorum sensing inhibitor (QSI), norharmane, that uniquely shows weak antibacterial activity but strongly inhibits pyocyanin production and biofilm formation of MDR P. aeruginosa. Biophysical experiments and molecular docking studies showed that norharmane competes with anthraniloyl-AMP for anthranilyl-CoA synthetase PqsA of P. aeruginosa at the ligand-binding pocket, which is not exploited by current inhibitors, thereby altering transcription regulatory activity. Moreover, norharmane exhibits synergy with polymyxin B. This synergism exhibits a high killing rate, low probability of resistance selection, and minimal cytotoxicity. Notably, norharmane can effectively boost polymyxin B activity against MDR P. aeruginosa-associated infections in animal models. Together, our findings provide novel insight critical to the design of improved PqsA inhibitors, and an effective combination strategy to overcome multiantibiotic bacterial resistance using conventional antibiotics and QSIs.

**IMPORTANCE** Pseudomonas aeruginosa is a dominant hospital-acquired bacterial pathogen typically found in immunocompromised individuals. It is particularly dangerous for patients with chronic lung diseases and was identified as a serious threat for patients in the 2019 Antimicrobial Resistance Threats report (https://www.cdc.gov/drugresistance/biggest-threats.html). In this study, we used activity-based high-throughput screening to identify norharmane, a potent and selective inhibitor of P. aeruginosa PqsA, which is a well-conserved master quorum sensing (QS) regulator in multidrug resistant (MDR) P. aeruginosa. This compound competitively binds anthranilyl-CoA synthetase PqsA at the anthraniloyl-AMP binding domain, which has not been exploited by known inhibitors. Remarkably, norharmane can significantly block the production of the virulence factor, pyocyanin (87%), and biofilm formation (80%) in MDR P. aeruginosa. Furthermore, norharmane is capable of augmenting polymyxin B activity against MDR P. aeruginosa in cell cultures and animal models. Taken together, these results suggest that norharmane may be an effective adjuvant for combating multiantibiotic bacterial resistance.

## INTRODUCTION

Quorum sensing (QS) is a form of bacterial communication that is crucial for the secretion of virulence factors, biofilm formation, and the production of public goods in Gram-negative and Gram-positive pathogens (e.g., P. aeruginosa, Vibrio harveyi, and *Staphylococci*) (1–5). Thus, interference with the QS circuit has the potential to be developed as a nontraditional antibacterial therapeutic option (6).

There are currently three well-established QS systems in P. aeruginosa, *las*, *rhl*, and *pqs*, that are interconnected and highly integrated (7–15). The three QS systems have been intensively investigated over the past decade. PqsA, a CoA-ligase enzyme, is the first synthase in the alkyl quinolone (AQ) biosynthetic pathway that blocks the production of virulence factors. Previous attempts to inhibit this enzyme are based on PqsA substrate analogs (e.g., halogenated anthranilate derivatives [8, 12, 16–18] and anthraniloyl-AMP analogs [19]), which decreases the level of the Pseudomonas quinolone signal (PQS) in P. aeruginosa. Despite their crucial role in PQS levels, these inhibitors are either weak or lacking in cellular activity, and their precise mechanisms of action remain unclear. As such, the substrate-based design has likely impeded PqsA inhibitor development efforts. A novel PqsA inhibitor, norharmane, was obtained based on high-throughput screening and has greatly expanded the development of quorum sensing inhibitors (QSIs). Moreover, we hypothesize that the potential clinical benefits for QSI therapies function synergistically with existing antibiotics and enhance the efficacy of antibiotics. However, this hypothesis lacks convincing evidence. Our findings demonstrate that QSIs in combination with antibiotics may represent a promising candidate to control outbreaks of drug-resistant bacteria.

In this study, we performed high-throughput screening of 3,000 deep-sea bacterial metabolites and identified norharmane as a potent PqsA inhibitor in multidrug resistant (MDR) P. aeruginosa. This compound triggers the dysregulation of the autoinduction response of the *pqs* QS system, leading to the inhibition of pyocyanin virulence and biofilm formation. Most notably, the synergistic activity between norharmane and multiple antibiotics was achieved, which can both reduce the amount of existing antibiotics to constrain their selective pressure and side effects, as well as extend the life span of antibiotics.

## RESULTS

### Discovery of QSI against MDR P. aeruginosa.

**FIG 1:**
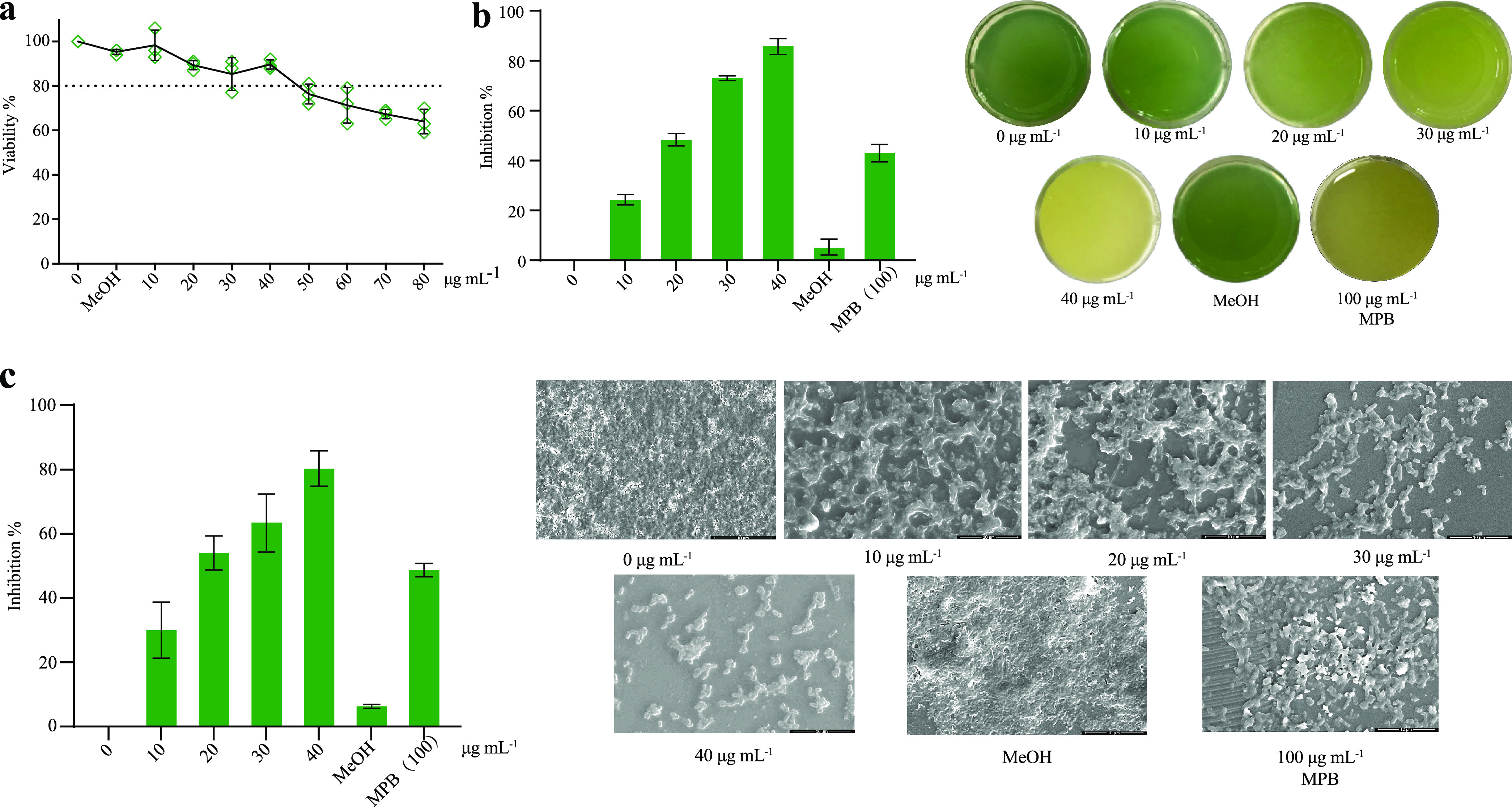
The biological activity of norharmane against MDR P. aeruginosa C218. (a) Effect of norharmane on the growth of MDR P. aeruginosa C218. (b) Effect of norharmane on pyocyanin production in MDR P. aeruginosa C218 under sub-MIC. (c) Effect of norharmane on biofilm formation in MDR P. aeruginosa C218 under sub-MIC. Individual data points (*n* = 3) and the mean ± SD are shown. MPB, 2-methyl-*N*-(2′-phenylethyl)butyramide.

We used MDR P. aeruginosa C218 as a screening tool to rapidly screen approximately 3,000 deep-sea bacteria provided by the Second Institute of Oceanography, SOA, China. Finally, norharmane was obtained from a deep-bacterium *Microbactrium* sp. 40DY182 based on activity screening. Norharmane is a class of aromatic β-carbolines and a normal body constituent from endogenous and external formation (20). The MIC of norharmane against P. aeruginosa C218 was>1 mg/mL; however, treatment with norharmane at concentrations varying from 10 to 40 μg mL^−1^ showed a no or little growth inhibitory effect compared to the carrier control, methanol (Fig. 1a). Within this concentration range, norharmane strongly inhibits pyocyanin production and biofilm formation in P. aeruginosa C218 with approximately 87% and 80% reduction, respectively, and is superior to the positive control, 2-methyl-*N*-(2′-phenylethyl)butyramide (21). These data demonstrated that norharmane was an effective QSI. Therefore, norharmane was selected for further investigation.

### Target determination of norharmane.

We performed microarrays to verify the norharmane functions following QS inhibition. The transcriptome data were submitted to the Genome Sequence Archive (GSA) with accession number CRA005738. P. aeruginosa treated with norharmane caused altered expression of many of the known QS-regulated genes (Fig. S1). For example, those phz operons encoding phenazine biosynthesis (e.g., *phzA1*-*A2*, *phzB1*-*B2*, *phzS*, *phzH*, and *phzM*) (7, 22) and other genes involved in the production of pyocyanin (e.g., *pqsE*) (23, 24) were significantly suppressed, confirming that the QS system of P. aeruginosa may represent a relevant *in vivo* target of norharmane (Fig. S2).

**FIG 2:**
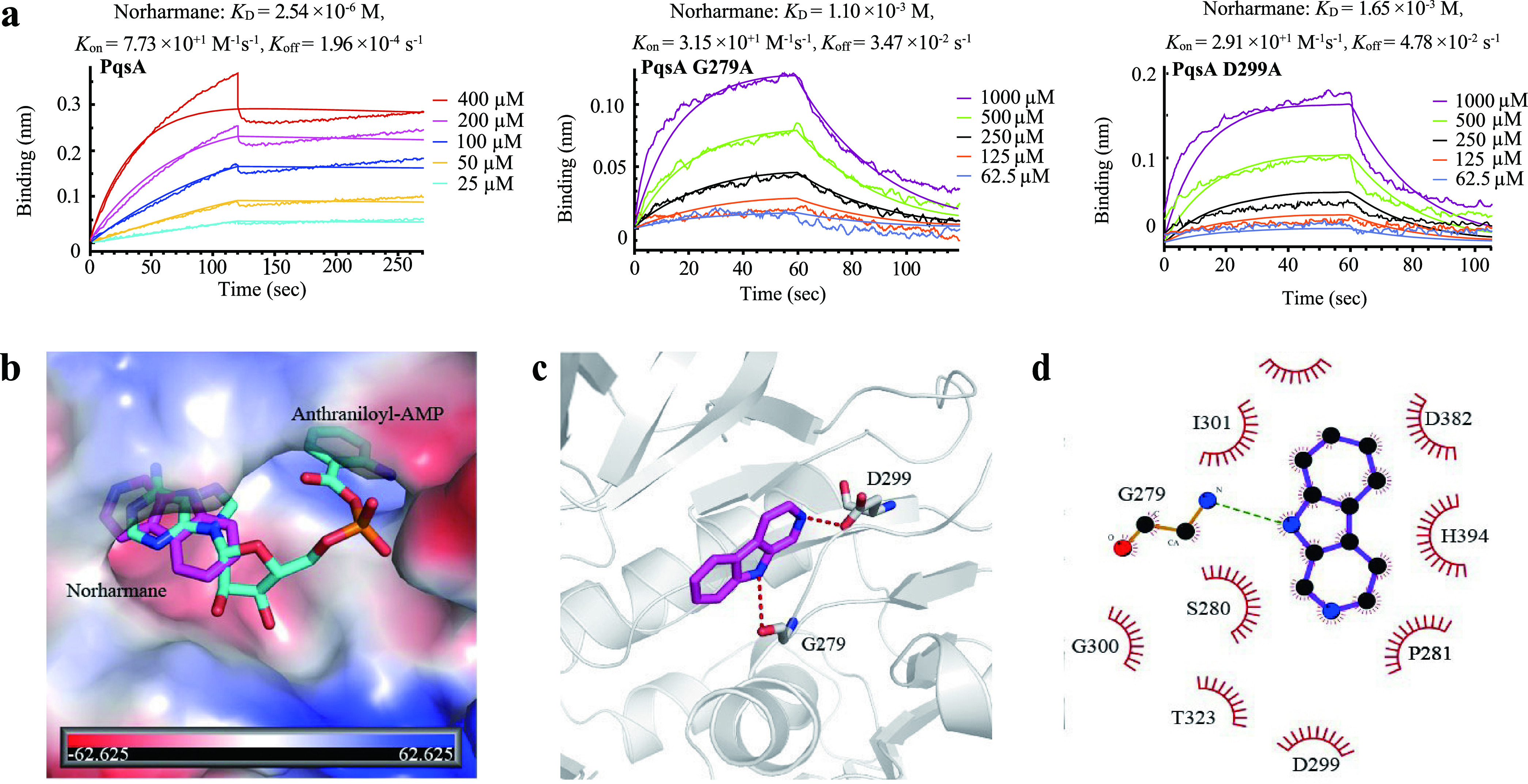
Norharmane targets PqsA protein. (a) Kinetic binding sensorgrams of norharmane and proteins (PqsA, PqsA G279A, and PqsA D299A). (b) Superimposition of the binding modes of norharmane and anthraniloyl-AMP from molecular docking analysis. Norharmane is shown as magenta sticks, and anthraniloyl-AMP is shown as cyan sticks, with the surface of PqsA depicted in vacuum electrostatics. (c) The predicted binding mode of norharmane. It is shown as magenta sticks, and key residues are shown as gray sticks. Hydrogen bonds are shown as a red dashed line. (d) Schematic diagram showing putative interactions between PqsA and norharmane. Residues involved in the hydrophobic interactions are denoted as starbursts, and hydrogen-bonding interactions are denoted by dotted green lines.

We used a label-free biolayer interferometric (BLI) to determine the dissociation constant (*K*_D_) of norharmane and QS-regulated proteins. Norharmane did not exhibit binding to LasI, RhlI, RhlR, PqsD, and PqsR, and exhibited weak binding to LasR and PqsE with *K*_D_ values of 2.29 mM and 0.5 mM, respectively (Fig. S3). However, norharmane showed strong binding to PqsA with *K*_D_ values of 2.54 μM (Fig. 2a), suggesting that PqsA was the most likely endogenous target of norharmane. To further show the molecular details of how norharmane interacts with PqsA, a molecular docking analysis was performed. As shown in Fig. 2b, norharmane occupies the binding pocket of the adenine ring in anthraniloyl-AMP, which is a reaction intermediate bound by PqsA, thereby leading to decreased biosynthesis of the quinolone quorum sensing factors, 4-hydroxy-2-heptylquinoline (HHQ) and PQS. The amino group in the indole ring of norharmane is hydrogen-bonded to the D299 side chain and the G279 nitrogen backbone (Fig. 2c). These two residues are highly conserved in other aryl-CoA ligases (Fig. S4). Furthermore, the compound forms extensive hydrophobic interactions with the surrounding residues (Fig. 2d). To validate the molecular modeling results, we performed the BLI binding assays with the norharmane and PqsA mutants, G279A and D299A, respectively. Consistent with the observed hydrogen bonds in the molecular docking study, G279 and D299 mutations to alanine significantly weakened the binding, which exhibited *K*_D_ values of 1.10 mM and 1.65 mM, respectively (Fig. 2a). Both G279 and D299 mutants showed a 433-fold and 650-fold decrease in the conversion rates, respectively. This molecular docking study combined with biophysical experiments indicated that the inhibition of PqsA by norharmane occurred through competition with anthraniloyl-AMP at the active site.

### Effect of norharmane on the P. aeruginosa AQ biosynthetic pathway, pyocyanin, and biofilms.

**FIG 3:**
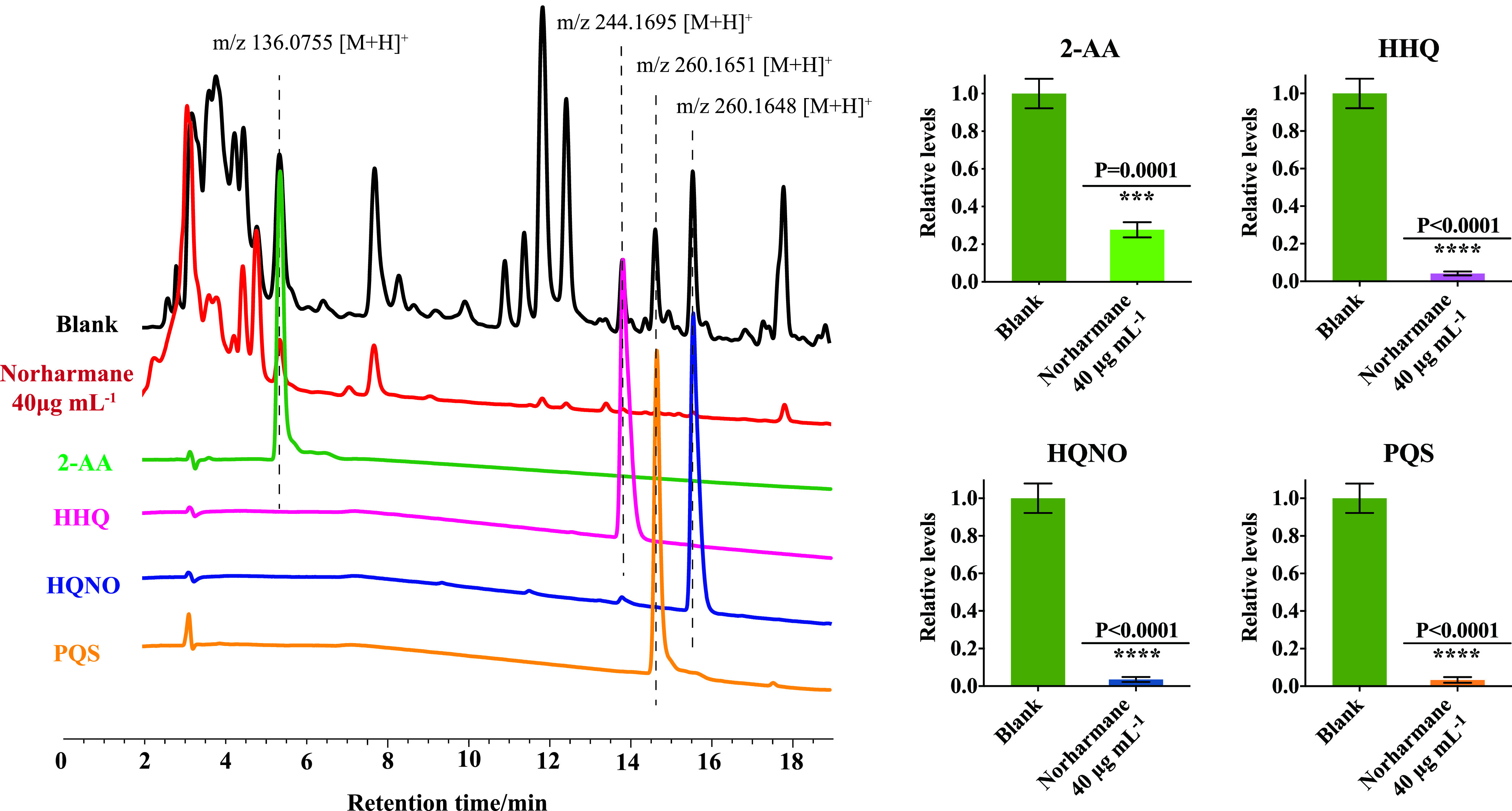
Relative quantification of 2-AA, HHQ, HQNO, and PQS was determined in P. aeruginosa C218 using reference standards and LC-MS/MS chromatograms. Blank is the untreated P. aeruginosa. Individual data points (*n* = 3) and mean ± SD are shown. *P* values were determined using an unpaired, two-tailed Student's *t* test. *, *P* < 0.05 versus the control; **, *P* < 0.01 versus the control; ***, *P* < 0.001 versus the control.

Next, we evaluated norharmane for its ability to inhibit HHQ, 4-hydroxy-2-heptylquinoline-N-oxide (HQNO), PQS, and 2-aminoacetophenone (2-AA) in P. aeruginosa C218. HHQ, HQNO, PQS, and 2-AA concentrations were determined by LC-MS/MS quantitation. Exposure to norharmane for 48 h caused a significant decrease in HHQ, HQNO, PQS, and 2-AA. A relative quantification analysis demonstrated that norharmane treatment at 40 μg mL^−1^ reduced HHQ, HQNO, PQS, and 2-AA by approximately 94%, 95%, 97%, and 72%, respectively (Fig. 3). Moreover, the transcriptome data showed that the expression of *pqsA*, *pqsB*, *pqsC*, *pqsD*, and *pqsE* genes of the AQ biosynthetic pathway were markedly inhibited in the norharmane-treated P. aeruginosa, further demonstrating that norharmane can effectively disrupt 2-AA, HHQ, HQNO, and PQS production (Fig. S5) (GSAaccession number CRA005738). Previous screens used to inhibit PqsA were based on PqsA substrate analogs, including halogenated anthranilate derivatives (8, 16–18) and anthraniloyl-AMP analogs (19). The most efficient inhibitor is 2-amino-6-fluorobenzoic acid (6FABA). Our results showed that norharmane inhibits 87% pyocyanin production and 80% biofilm formation, and is superior to the positive control, 2-amino-6-fluorobenzoic acid (Fig. S6).

### Norharmane potentiates antibiotics against MDR P. aeruginosa.

**FIG 4:**
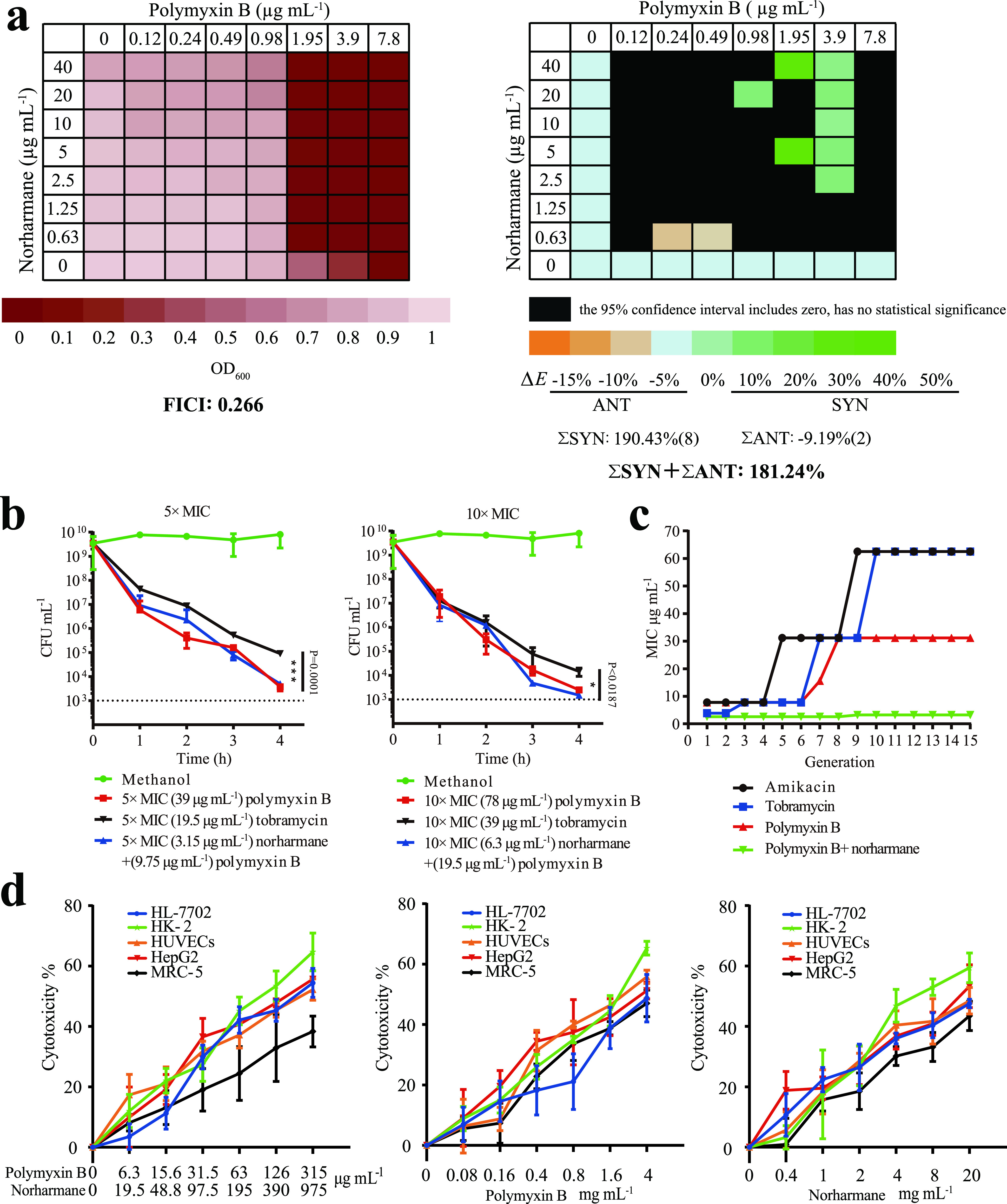
Norharmane-polymyxin B exhibits synergism, rapid-killing kinetics, as well as lower development of resistance and cytotoxicity. (a) The synergism between norharmane and polymyxin B was evaluated against P. aeruginosa C218 by FICI and *ΔE*. (b) P. aeruginosa C218 was treated with polymyxin B, tobramycin, and norharmane-polymyxin B. Bacterial CFU counts were calculated by serial dilution and followed by drop plating onto LB plates. Individual data points (*n* = 3) and mean ± SD are shown. *P* values were determined using an unpaired, two-tailed Student's *t* test. *, *P* < 0.05 versus the control; **, *P* < 0.01 versus the control; ***, *P* < 0.001 versus the control. (c) Development of P. aeruginosa C218 resistance to amikacin, tobramycin, and polymyxin B was evaluated by MIC. Development of P. aeruginosa C218 resistance to norharmane-polymyxin B was evaluated by FICI. (d) Cytotoxicity of HL-7702, HK-2, HUVECs, HepG2, and MRC-5 cells treated with norharmane, polymyxin B, or norharmane in combination with polymyxin B was tested based on MTT assay.

To explore the clinical benefits of norharmane, we performed a checkerboard assay to determine the potency of norharmane used in combination with 20 existing antibiotics used to treat P. aeruginosa. As shown in Fig. 4a, norharmane treatment exhibited synergism with polymyxin B, with 0.266 FICI (fractional inhibitory concentration index) and 181.24% *ΔE* indexes (interaction between the predicted and measured percentage of growth at various concentrations). This suggests that the norharmane-polymyxin B combination exhibited increased antimicrobial activity against P. aeruginosa C218. In addition to polymyxin B, norharmane synergistically interacted with imipenem and cilastatin sodium and levofloxacin *in vitro* (Fig. S7).

A killing assay revealed that norharmane-polymyxin B combination exhibited equivalent bactericidal activity as treatment with polymyxin B alone and was superior to tobramycin (an antibiotic associated with P. aeruginosa C218 sensitivity); however, the required dosage of polymyxin B was significantly lower (Fig. 4b). In a panel of MDR clinical strains of P. aeruginosa (Table S1), the synergistic combination also exhibited equivalent bactericidal activity compared with polymyxin B alone (Fig. S8). The MICs were 1.95 μg mL^−1^ for polymyxin B plus 0.63 μg mL^−1^ norharmane for MDR P. aeruginosa, with 3.9–15.6 μg mL^−1^ of MICs for polymyxin B alone (Table S2).

An important unresolved issue was whether the norharmane-polymyxin B combination could be employed sequentially to select against resistance. Amikacin and tobramycin were selected as the positive control due to their sensitivity to P. aeruginosa C218 (MIC 7.8 μg mL^−1^ and 3.9 μg mL^−1^). The preliminary results indicated that amikacin, tobramycin, and polymyxin B generated strains were 16-fold, 16-fold, and 8-fold resistant after 15 passages, respectively (Fig. 4c), whereas serial passaging in the norharmane-polymyxin B combination was associated with a minimal increase in resistance.

We further evaluated the cytotoxicity of norharmane-polymyxin B on human cell lines. The synergistic combination was relatively nontoxic, exhibiting an IC_50_ of greater than or equal to 126 μg mL^−1^ of norharmane plus 390 μg mL^−1^ polymyxin B against human hepatoma HepG2 cells, human liver hepatocellular HK-2 cells, human umbilical vein endothelial HUVECs cells, human liver HL-7702 cells, and human lung fibroblast MRC-5 cells (Fig. 4d).

### Norharmane promotes antibiotic potency in a murine infection model.

**FIG 5:**
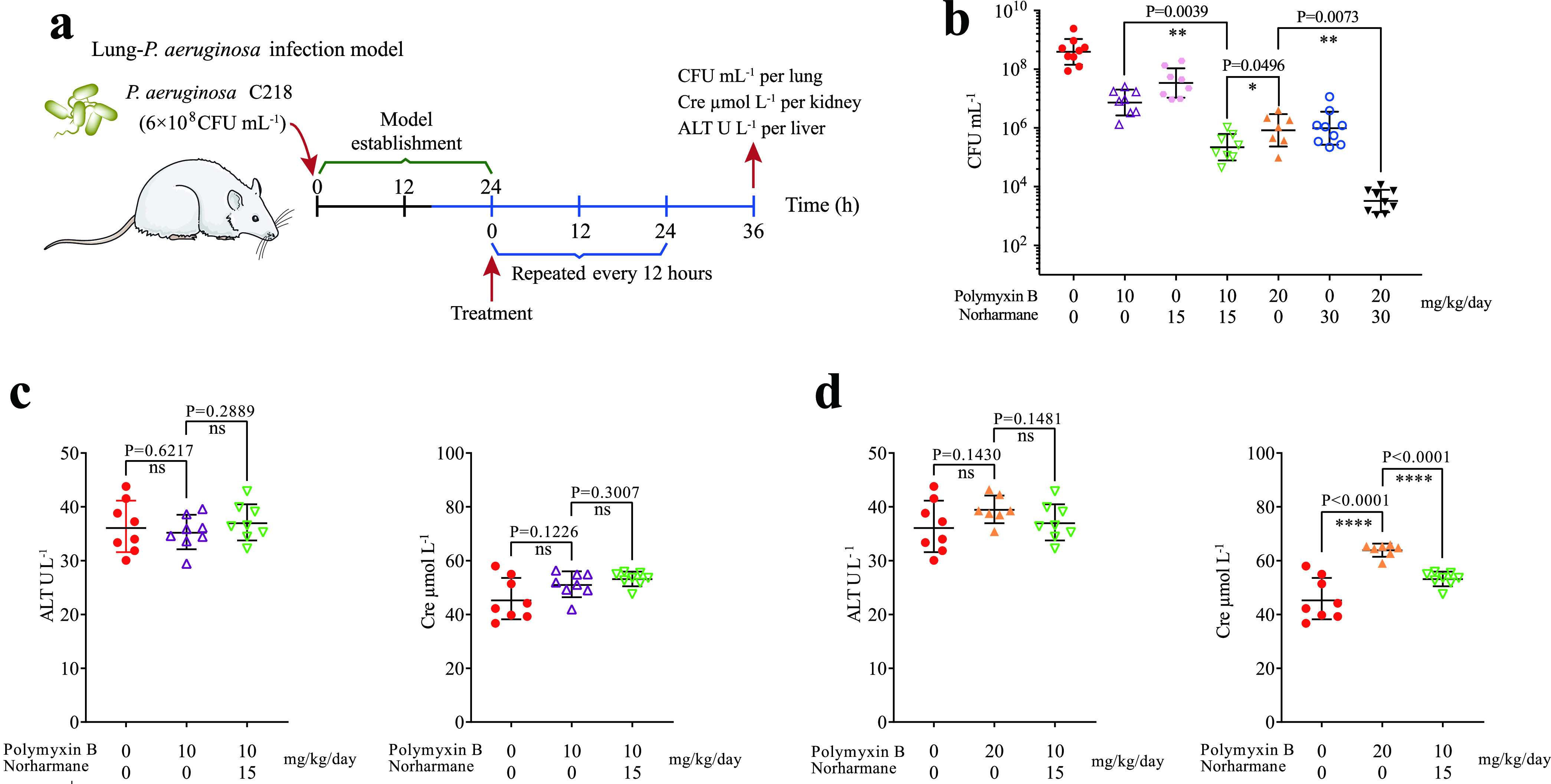
*In vivo* efficacy of norharmane-polymyxin B in a mouse lung P. aeruginosa C218 infection model. (a) Scheme of the experimental protocol for the mouse lung P. aeruginosa infection model. (b) Synergism between norharmane and polymyxin B was evaluated in a mouse lung P. aeruginosa C218 infection model. At 36 h after treatment, the mice were euthanized, and their infected lungs were aseptically excised. The bacterial loads (Log_10_ CFU of P. aeruginosa C218) in the lung were counted. The mean ± SD for each experimental group is shown. (c–d) Evaluation of nephrotoxicity and hepatotoxicity. Cre (creatine) and ALT (alanine aminotransferase) were analyzed. The mean ± SD for each experimental group is shown. *P* values were determined using an unpaired, two-tailed Student's *t* test; ns, *P* > 0.05 versus the control; **, *P* < 0.01 versus the control; ***, *P* < 0.001 versus the control; ****, *P* < 0.0001 versus the control.

**FIG 6:**
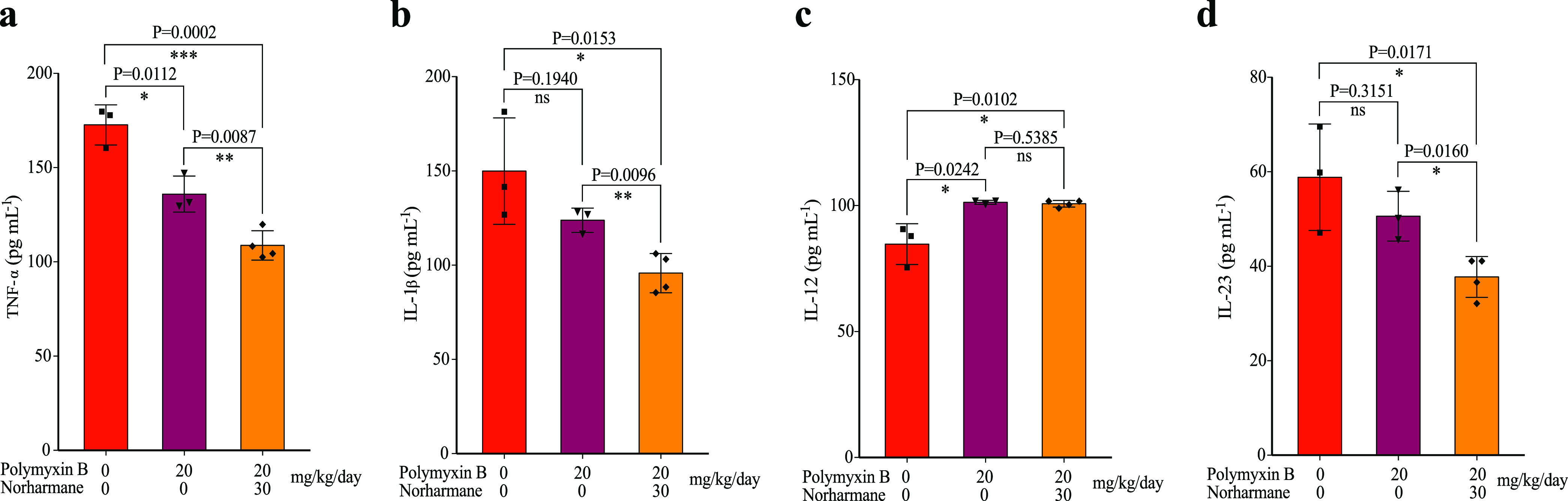
Inflammatory cytokine expression of TNF-α (a), IL-1β (b), IL-12 (c), and IL-23 (d) was determined by ELISA. The data shown are means ± SD. *P* values were determined using an unpaired, two-tailed Student's *t* test; ns, nonsignificant; *, *P* < 0.05 versus the control; **, *P* < 0.01 versus the control.

*In vivo* efficacy of the combination therapy was evaluated using a mouse lung MDR P. aeruginosa infection model, which mimics a human lung infection (Fig. 5a). The norharmane-polymyxin B combination reduced the bacterial abundance in the infected tissue by approximately 1–2 orders of magnitude (Fig. 5b). Treatment with 15 mg/kg/day of norharmane in combination with 10 mg/kg/day of polymyxin B and 30 mg/kg/day of norharmane in combination with 20 mg/kg/day of polymyxin B resulted in approximately 31-fold and 245-fold decreases in the bacterial burden compared with 10 mg/kg/day of polymyxin B and 20 mg/kg/day of polymyxin B, respectively. This finding suggests that the dosage of norharmane affected the bacterial counts under synergistic interactions; however, norharmane exhibited stronger synergy with the concomitant increase in the effective dose. This finding is consistent with the observation that the coadministration of polymyxin B and norharmane *in vitro* exhibited stronger synergistic bactericidal activity with the increasing dosage of norharmane. Moreover, norharmane supplementation (15 mg/kg/day norharmane + 10 mg/kg/day polymyxin B) did not show increased cytotoxicity in the kidney and liver of the mice compared with polymyxin B treatment alone (10 mg/kg/day of polymyxin B) (Fig. 5c and d). Notably, the use of 15 mg/kg/day norharmane in combination with 10 mg/kg/day of polymyxin B resulted in approximately 4-fold (*P* = 0.0496) decrease in bacterial burden compared with 20 mg/kg/day polymyxin B treatment alone (Fig. 6b). However, there was an obvious decrease in renal damage, superior to polymyxin B (20 mg/kg/day) treatment alone (Fig. 5d). This suggests that norharmane-polymyxin B could significantly decrease renal damage compared with polymyxin B alone while achieving the same bactericidal effect. This helps to alleviate the nephrotoxicity caused by polymyxin B.

Many cytokines are involved in the development of inflammation caused by bacterial infections (25). To understand which cytokines are implicated in the inhibition by tissue damage in the mouse lung of a P. aeruginosa infection model, we assessed several cytokines isolated from infected mouse lungs. The results revealed a decreased expression of cytokines, including TNF-α, IL-1β, and IL-23. Following treatment, the combination of norharmane and polymyxin B significantly decreased TNF-α, IL-1β, and IL-23 compared with polymyxin B alone in the infected model (Fig. 6a to d). However, the synergistic combination had no impact on IL-12 compared with polymyxin B treatment alone (Fig. 6c). TNF-α, IL-1β, and IL-23 were found to be highly pathogenic and could induce pathogenic T_H_17 helper (T_H_17) cells, promoting T_H_17 cell immune responses that aggravate inflammatory diseases (26, 27). The inflammatory cytokine, IL-12, could promote a type 1 T helper (T_H_1) immune response and aggravate tissue damage (25). These results confirmed that norharmane might increase the susceptibility of polymyxin B to impair pathogenic T_H_17 cells, thereby decreasing tissue damage.

## DISCUSSION

**FIG 7:**
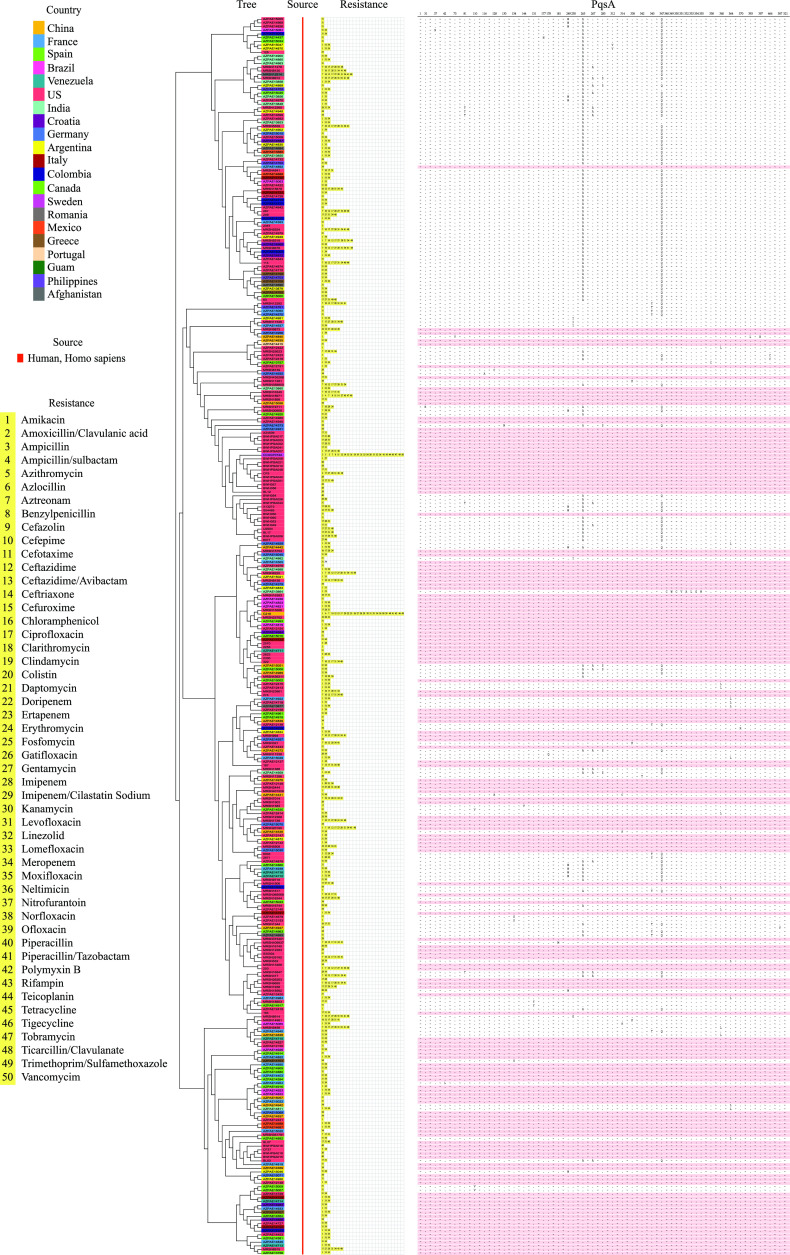
Distribution and characteristics of the PqsA protein from 335 drug-resistant P. aeruginosa. The whole-genome sequences of 334 drug-resistant P. aeruginosa used to construct phylogenetic trees were obtained from a publicly available genomic data bank (https://patricbrc.org/). The whole-genome sequence of MDR P. aeruginosa C218 was obtained from our lab and has been submitted to the NCBI Short Read Archive under accession number SRR11939661. The tree was generated by CVTree 3.0 (http://cvtree.online/v3/cvtree/) using a composition vector (CV) approach. The PqsA sequence was aligned using ClustalW. The same amino acids were omitted and are represented with “~”.

Although the observations of the present study illustrate that PqsA represents a potent target for controlling biofilm formation and the production of other virulence factors, little is known about PqsA mutations at the amino-acid sequence level. Recently, hundreds of reference genomes of drug-resistant P. aeruginosa from humans (334 identified drug-resistant P. aeruginosa) have become available in the PATRIC database (https://patricbrc.org/). Over 98.46% of the PqsA amino acid sequences of all 334 drug-resistant P. aeruginosa are consistent with that of MDR P. aeruginosa C218 (GSA accession number: CRA005729) (Fig. 7; Fig. S9; and Table S3). Among these, 156 strains (approximately 46.70% of all drug-resistant P. aeruginosa) were completely identical at the amino-acid sequence level. Notably, all P. aeruginosa have the same anthraniloyl-AMP binding domain in PqsA, suggesting that the ligand-binding pocket of PqsA can be considered as a universal target for the identification of therapeutic agents against drug-resistant P. aeruginosa. This finding will facilitate the further modification and optimization of norharmane to improve the binding affinity, tune efficacy, and possibly broaden its use beyond initial expectations.

One main barrier to QSI development will be to demonstrate that their combination therapy with antibiotics has a clear clinical benefit (8, 28). Traditional detection methods of combination therapy based on MIC and an MIC checkerboard assay of antibiotics cannot be adapted to evaluate the coadministration of anti-virulence drugs and antibiotics, since antivirulence drugs do not effectively inhibit growth or kill pathogens. Thus, alternative *in vitro* detection methods are developed using the MIC of antibiotics and sub-MIC of anti-virulence drugs. Moreover, the *in vitro* efficacy of the norharmane-polymyxin B combination against P. aeruginosa provides an opportunity to explore *in vivo* activity. The *in vivo* superiority of combination therapy versus standalone antibiotic therapy will be the most convincing method of confirming a clinical value of an add-on therapy. A key finding from our work is that *in vivo* treatment with norharmane-polymyxin B both significantly decreases the CFU on implants but also obviously reduces nephrotoxicity due to the decreased usage of polymyxin B. This greatly solves the most problematic side effect of polymyxin B therapy: nephrotoxicity. Further research should examine whether such a synergistic combination can be used repeatedly and maintained over longer periods of time *in vivo* without the appearance of mutations that confer increasing resistance and diminish norharmane-polymyxin B synergy. Additionally, whether norharmane-polymyxin B combination can generally outperform conventional antibiotic combinations in clinical infections also remains to be established. This study has made the first step in these directions. Our results warrant further development of synergistic QSI antibiotics as potential strategy for difficult-to-treat infections caused by antibiotic-resistant pathogens.

Given the weak antibacterial activity of norharmane, it is likely that the synergy observed *in vivo* is due partly as a result of norharmane antivirulence activity, as the dose-response potentiation is observed under a sub-MIC of norharmane that does not impair bacterial growth. We speculate that the bacterial biofilm damaged by norharmane may increase the passive diffusion of polymyxin B, which consequently led to the improved activity. Consistent with this conclusion, the biofilm-defective PA14 *pqsA* transposon mutant appeared to have a greater distribution in a single bacterial cell state rather than the biofilm state in the mouse models and was more susceptible to clearance by ciprofloxacin compared to the PA14 wild-type strain (10). However, further studies are required to elucidate the molecular mechanisms underlying norharmane and polymyxin B synergy in greater detail.

In conclusion, norharmane was identified as a potent QSI against the virulence of MDR P. aeruginosa. This finding demonstrates that norharmane has the potential to be developed into a clinically useful nontraditional antibacterial agent. This is supported by the activities of combined norharmane and polymyxin B both *in vitro* and *in vivo* against clinically isolated MDR P. aeruginosa, particularly for P. aeruginosa that is resistant to β-lactam combination agents. This latter observation is particularly important because MDR P. aeruginosa frequently exhibits resistance to different classes of antibacterial agents. Undoubtedly, treatment of norharmane as a potent QSI combined with antibiotics can circumvent MDR P. aeruginosa and shows a great potential in clinic application.

## MATERIALS AND METHODS

### Bacterial strains.

*Microbactrium* sp. 40DY182 was isolated from the upper 5,591 m of the Western Pacific Ocean. MDR P. aeruginosa was provided by the Second Affiliated Hospital of Zhejiang University School of Medicine and Zhejiang Provincial People’s Hospital (Hangzhou, China).

### Extraction and isolation.

A total of 80 L of bacterial cultures was extracted with ethyl acetate. Norharmane was finally obtained using the activity tracking method. See the supplemental material for the detailed methods.

### Growth curves.

The overnight culture of P. aeruginosa C218 was diluted 1:10,000 and incubated with norharmane for 18 h at 37°C, and the OD_600_ was measured.

### Pyocyanin and biofilm assay.

We used a previously described method to measure pyocyanin and P. aeruginosa C218 biofilms (21).

### Protein expression and purification.

The codons coding for amino acids of *LasI*, *RhlI*, *RhlR*, *PqsA*, *PqsA^G279A^*, *PqsA^D299A^*, *PqsD*, *PqsR*, *LasR*, and *PqsE* genes were optimized through the MaxCodon Optimization Program (V13) and our optimization software, respectively. These genes were inserted into the expression vector by the whole gene synthesis technologies. All of the constructs were verified by automated DNA sequencing. Recombinant proteins were produced in E. coli strain BL21 Star (DE3) transformed with *pET30a-His_6_-lasI*, *pET30a-His_6_-rhlI*, *pET30a-His_6_-rhlR*, *pET30a-His_6_-pqsA*, *pET30a-His_6_-pqsA^G279A^*, *pET30a-His_6_-pqsA^D299A^*, *pET30a-His_6_*-*pqsD*, *pET30a-His_6_*-*pqsR*, *pET30a-His_6_*-*LasR*, and *pET30a-His_6_*-*pqsE*, respectively. The recombinant E. coli strain was cultured in LB media containing 50 μg mL^−1^ kanamycin. Cells were cultivated in baffled flasks until reaching a density (OD_600_) of 0.5~0.8 and then induced with 0.1–0.2 mM isopropyl β-d-thiogalactopyranoside for 16–24 h at 15°C. Cells were resuspended and sonicated in 50 mM Tris buffer, pH 8.0 containing 300 mM NaCl, and 20 mM imidazole supplement with 1% Triton X-100, 1 mM Dithiothreitol (DTT), and 1 mM phenylmethanesulfonyl fluoride (PMSF). Cell debris was removed by centrifugation. Ni-NTA was equilibrated twice using 50 mM Tris buffer, pH 8.0, 300 mM NaCl, and 20 mM imidazole. The His_6_-tagged proteins were purified by immobilized metal ion affinity chromatography with nickel-iminodiacetic acid resin (Ni-IDA) equilibrated with a wash buffer containing 50 mM Tris buffer, pH 8.0 containing 300 mM NaCl, and 20–500 mM imidazole supplement with 1% Triton X-100, 1 mM DTT, and 1 mM PMSF. The purified protein was assessed by SDS-PAGE.

### Biolayer interferometry assay.

The interaction between proteins and norharmane was quantified by biolayer interferometry (BLI) using on an Octet K2 system (Pall ForteBio, Menlo Park, CA, USA). Ni-NTA Biosensors (ForteBio, part nos. 18-5102, 18-5101, and 18–101) and Aminopropylsilane Biosensors (ForteBio, part no. 18-5046) were prewetted with buffer (PBS pH 7.4, 0.05% Tween 20, 5% DMSO) to establish a stable baseline prior to immobilization. Biotinylated protein targets were then immobilized onto Ni-NTA Biosensors or Aminopropylsilane Biosensors.

### QS signal measurements.

P. aeruginosa C218 were inoculated into LB medium in the presence or absence of norharmane and cultured at 37°C for 48 h. The supernatant was extracted three times with equal volumes of acidified ethyl acetate. The resultant organic layer was evaporated, and the residues were dissolved in methanol. Liquid chromatography-tandem mass spectrometry (LC-MS/MS) was adopted for HHQ, HQNO, PQS, and 2-AA quantification.

### MIC assay.

MIC determination using the standard micro-dilution method was performed according to the Clinical and Laboratory Standards Institute (29). The concentration of each antibiotic that can completely inhibit visible growth was defined as its MIC. Experiments were performed in triplicate.

### Cytotoxicity.

MTT tests were used to determine the level of toxicity. See the supplemental material for the detailed methods.

### Antibiotic synergy test.

A microdilution checkerboard assay was used to determine the synergy of norharmane with clinical antibiotics against P. aeruginosa. See the supplemental material for the detailed methods.

### Time-dependent killing.

P. aeruginosa was incubated at 37°C, with shaking at 180 rpm for 17 h until the concentration reached approximately 4 × 10^9^ CFU mL^−1^. The cells were then diluted in 1:100 in fresh LB medium. Next, 200 μL cell culture was added to the wells of a 96-well assay, block-treated with 5× MIC antibiotics or 10× MIC antibiotics, and incubated at 37°C with shaking at 180 rpm for 4 h. Every hour, the colonies were counted to enumerate the number of cells. Experiments were conducted in triplicate. *P* values were determined using an unpaired, two-tailed Student's *t* test.

### Resistance evaluation.

Stationary-phase P. aeruginosa C218 cells were transferred into fresh LB medium containing 3.9 μg mL^−1^ amikacin (1/2 MIC), 1.95 μg mL^−1^ tobramycin (1/2 MIC), 3.9 μg mL^−1^ polymyxin B (1/2 MIC), and combination of polymyxin B (0.32 μg mL^−1^) and norharmane (0.98 μg mL^−1^) (1/2 MIC) at 37°C for 48 h, respectively. Serial passaging was performed based on the above method for 7 passages. After the 7th passage, stationary-phase cells were transferred into fresh LB medium containing 7.8 μg mL^−1^ amikacin (MIC), 3.9 μg mL^−1^ tobramycin (MIC), 7.8 μg mL^−1^ polymyxin B (MIC), and combination of polymyxin B (0.63 μg mL^−1^) plus norharmane (1.95 μg mL^−1^) (MIC) at 37°C for 48 h, respectively. For every generation, the MICs were tested in relation to amikacin, tobramycin, polymyxin B, and norharmane-polymyxin B treatment against P. aeruginosa C218, respectively.

### Mouse lung infection model used to evaluate drug efficacy.

After establishing the mouse lung infection model, mice were treated with polymyxin B, norharmane, or norharmane-polymyxin B. See the supplemental material for the detailed methods.

### Cytokine and chemokine profiling.

After the mice had been humanely euthanized, their lungs were lavaged four times with 2 mL normal saline. The retained bronchoalveolar lavage fluid was centrifuged, and the recovered supernatants were collected and stored at −80°C. The level of IL-12, TNF-α, IL-1β, and IL-23 expression was detected by an ELISA, in accordance with the manufacturer’s instructions (R&D Systems).

### Transcriptome analysis of P. aeruginosa.

RNA preparation of RNA-sequencing libraries and deep-sequencing were performed by Novogene Co., Ltd. (Tianjing, China).

### Data availability.

Source data supporting the findings of the present study are included in the article. The raw sequence data reported in this article have been deposited in the Genome Sequence Archive (Genomics, Proteomics & Bioinformatics 2021) in the National Genomics Data Center (Nucleic Acids Res 2021), China National Center for Bioinformation/Beijing Institute of Genomics, Chinese Academy of Sciences (GSA: CRA005738; CRA005729); they are publicly accessible at https://ngdc.cncb.ac.cn/gsa.
