# Supplementary material for: A Small-Molecule Inhibitor of the Anthranilyl-CoA Synthetase PqsA for the Treatment of Multidrug-Resistant Pseudomonas aeruginosa
Source: Microbiol Spectr. 2022 Jul 20;10(4):e02764-21. doi: 10.1128/spectrum.02764-21 (PMC9430567; doi:10.1128/spectrum.02764-21)
Supplement: Supplemental file 1 — Supplemental material. Download spectrum.02764-21-s0001.pdf, PDF file, 1.7 MB [file spectrum.02764-21-s0001.pdf]

Supplemental Material For:

A small-molecule inhibitor of the anthranilyl-CoA synthetase PqsA for the treatment of multidrug-resistant *Pseudomonas aeruginosa*

Jianwei Chen[1,†], Yaojia Lu[1,†], Fei Ye[2†], Hongfang Zhang[1], Yonglie Zhou[3], Jiangtao Li[4], Qiang Wu[5],Xuewei Xu[6], Qihao Wu[1], Bin Wei[1], Huawei Zhang[1]*, Hong Wang[1]*

[1]College of Pharmaceutical Science & Collaborative Innovation Center of Yangtze River Delta Region Green Pharmaceuticals, Zhejiang University of Technology, Hangzhou, China. [2]College of Life Sciences, Zhejiang Sci-Tech University, Hangzhou, China. [3]Zhejiang Provincial People's Hospital, Hangzhou, China. [4]The Second Affiliated Hospital of Zhejiang University School of Medicine, Hangzhou, China. [5]State Key Laboratory of Quality Research in Chinese Medicines, Macau University of Science and Technology, Macau, China. [6]Second Institute of Oceanography, MNR, Hangzhou, China.

Table S1. Clinical *P. aeruginosa* isolated hospitals

No.	*P. aeruginosa*	Antibiotic type	Resistant antibiotic	Drug resistance
1	53482	β-lactam combination agents	Piperacillin/Tazobactam	MDR
		Carbapenems	Imipenem	
			Meropenem	
		Aminoglycosides	Tobramycin	
			Gentamicin	
			Amikacin	
		Cephems	Ceftazidime	
			Cefepime	
			Cefoperazone/Sulbactam	
		Fluoroquinolones	Ciprofloxacin	
			Levofloxacin	
		Phosphonic acids	Fosfomycin	
		Lipopeptides	Polymyxin B	
2	554	Penicillins	Piperacillin	MDR
		β-lactam combination agents	Amoxicillin/clavulanic acid	
		Carbapenems	Imipenem	
		Aminoglycosides	Amikacin	
			Gentamicin	
		Cephalosporins	Cefotaxime	
		Sulfonamides	Trimethoprim-sulfoxazole	
		Fluoroquinolones	Moxifloxacin	
3	569	Penicillins	Piperacillin	MDR
		β-lactam combination agents	Amoxicillin/clavulanic acid	
			Piperacillin/Tazobactam	
		Cephalosporins	Cefotaxime	
			Cefoperazone/Sulbactam	
		Sulfonamides	Trimethoprim-sulfoxazole	
4	484	Penicillins	Piperacillin	MDR
		β-lactam combination agents	Amoxicillin/clavulanic acid	
		Aminoglycosides	Amikacin	
			Gentamicin	
		Cephalosporins	Cefotaxime	
		Sulfonamides	Trimethoprim-sulfoxazole	
		Fluoroquinolones	Ciprofloxacin	
			Moxifloxacin	
5	778	Penicillins	Piperacillin	MDR
		β-lactam combination agents	Ticarcillin/clavulanate	
		Cephems	Ceftazidime	
		Fluoroquinolones	Levofloxacin	
			Ofloxacin	
6	779	Penicillins	Piperacillin	MDR
		β-lactam combination agents	Imipenem	
		Cephems	Ceftazidime	

Continued

No.	*P. aeruginosa*	Antibiotic type	Resistant antibiotic	Drug resistance
7	782	Penicillins	Piperacillin	MDR
		β-lactam combination agents	Ticarcillin/clavulanate	
		Aminoglycosides	Tobramycin	
			Gentamicin	
			Amikacin	
			Netilmicin	
		Cephems	Ceftazidime	
			Cefoperazone/Sulbactam	
8	801	Penicillins	Piperacillin	MDR
		Monobactams	Aztreonam	
		β-lactam combination agents	Piperacillin/Tazobactam	
			Ticarcillin/clavulanate	
		Cephems	Cefoperazone/Sulbactam	
			Cefepime	
			Ceftazidime	
		Fluoroquinolones	Levofloxacin	
			Ofloxacin	
		Lipopeptides	Polymyxin B	
9	803	Penicillins	Piperacillin	MDR
		Carbapenems	Imipenem	
		β-lactam combination agents	Piperacillin/Tazobactam	
			Ticarcillin/clavulanate	
		Aminoglycosides	Tobramycin	
			Gentamicin	
			Netilmicin	
		Cephems	Ceftazidime	
			Cefepime	
		Fluoroquinolones	Levofloxacin	
			Ciprofloxacin	
			Norfloxacin	
			Ofloxacin	
10	805	Penicillins	Piperacillin	MDR
		Carbapenems	Imipenem	
		β-lactam combination agents	Ticarcillin/clavulanate	
		Aminoglycosides	Tobramycin	
			Gentamicin	
			Netilmicin	
		Cephalosporins	Ceftazidime	
		Fluoroquinolones	Levofloxacin	
			Ciprofloxacin	
			Norfloxacin	
			Ofloxacin	

TABLE S2 MICs of polymyxin B in 10 clinical MDR *P. aeruginosa*

No.	Strains	Minimal inhibitory concentration (MIC)
		Polymyxin B μg mL^{-1}
1	MDR *P. aeruginosa* 53482	15.6
2	MDR *P. aeruginosa* 554	3.9
3	MDR *P. aeruginosa* 569	3.9
4	MDR *P. aeruginosa* 484	7.8
5	MDR *P. aeruginosa* 778	7.8
6	MDR *P. aeruginosa* 779	7.8
7	MDR *P. aeruginosa* 782	7.8
8	MDR *P. aeruginosa* 801	15.6
9	MDR *P. aeruginosa* 803	7.8
10	MDR *P. aeruginosa* 805	7.8

TABLE S3 Minimum inhibitory concentration (μg mL^{-1}, MIC) of clinical antibiotics used to treat *P. aeruginosa* against MDR *P. aeruginosa* C218

Strain	Class	Antibiotic	MIC
MDR *P. aeruginosa* C218	Penicillins	Piperacillin	4000
		Azlocillin	4000
	β-lactam combination agents	Ceftazidime-avibactam	62.5
		Piperacillin-tazobactam	500
		Ticarcillin-clavulanate	32000
		Imipenem and cilastatin sodium	2000
	Cephems	Cefepime	4000
		Ceftazidime	500
	Monobactams	Aztreonam	4000
	Carbapenems	Meropenem	500
		Doripenem	500
		Imipenem	500
	Lipopeptides	Polymyxin B	7.8
		Colistin	3.9
	Aminoglycosides	Gentamycin	9.75
		Amikacin	7.8
		Neltimicin	31.25
		Tobramycin	3.9
		Kanamycin	310
	Fluoroquinolones	Levofloxacin	62.5
		Ciprofloxacin	15.6
		Ofloxacin	500
		Norfloxacin	4000
		Lomefloxacin	125
		Gatifloxacin	62.5

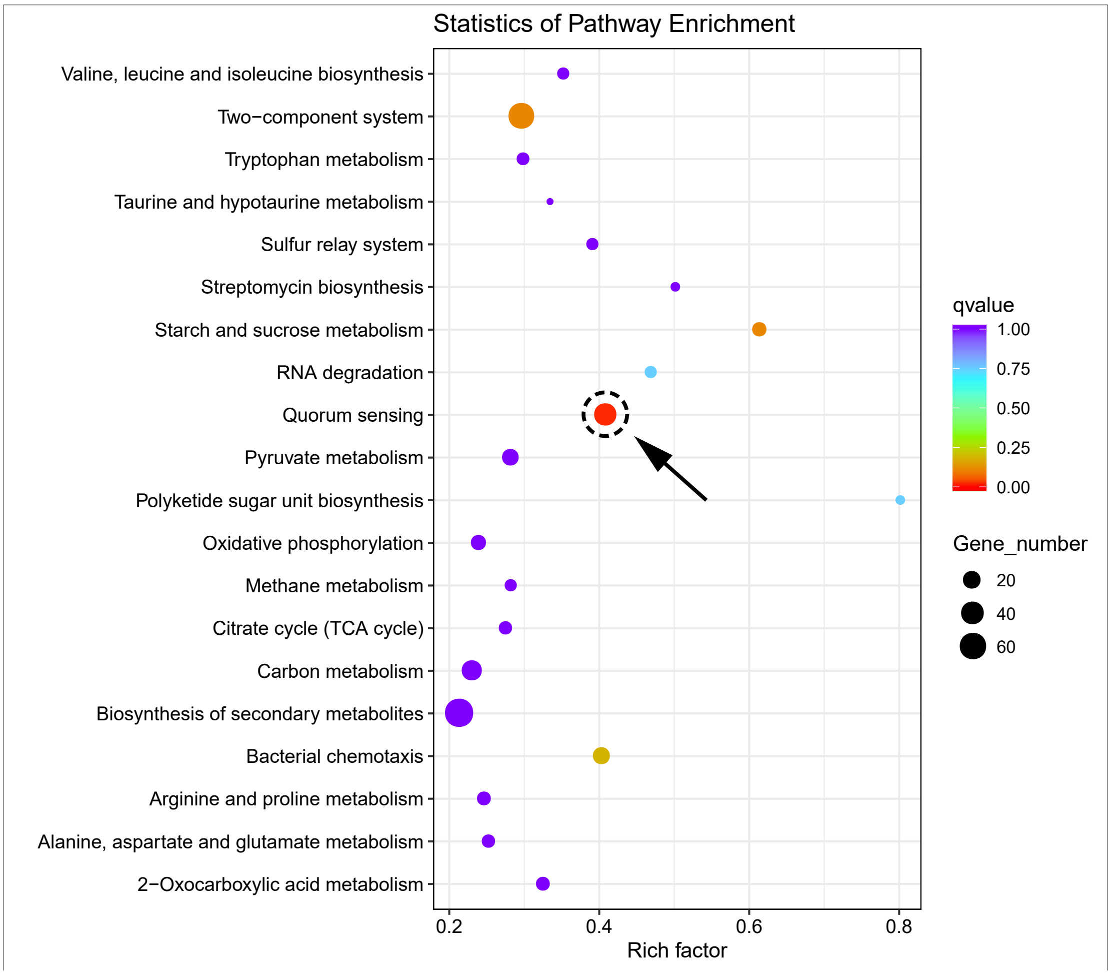

FIG S1 Classifications of downregulated genes in the *P. aeruginosa* +norharmane group compared with the *P. aeruginosa* group based on Kyoto Encyclopedia of Genes and Genomes (KEGG) analysis.

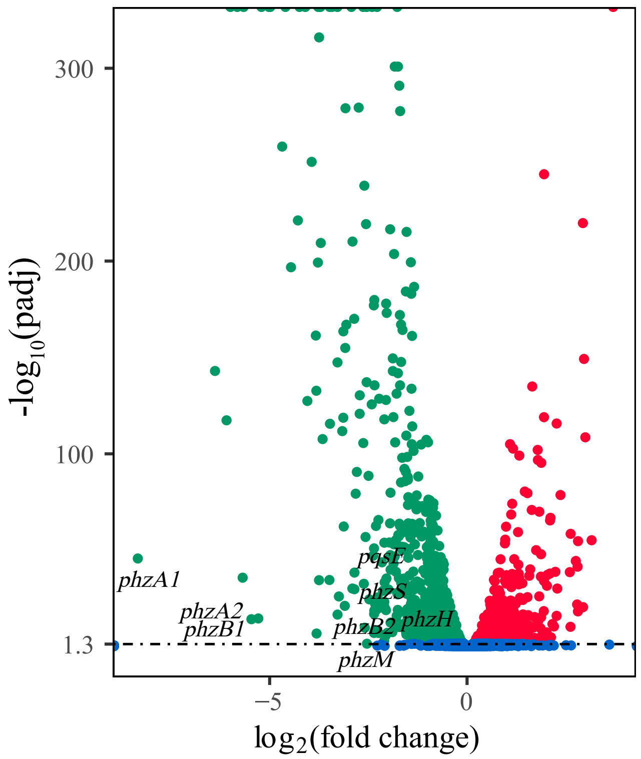

FIG S2. Transcriptome analysis of norharmane-treated *P. aeruginosa*.

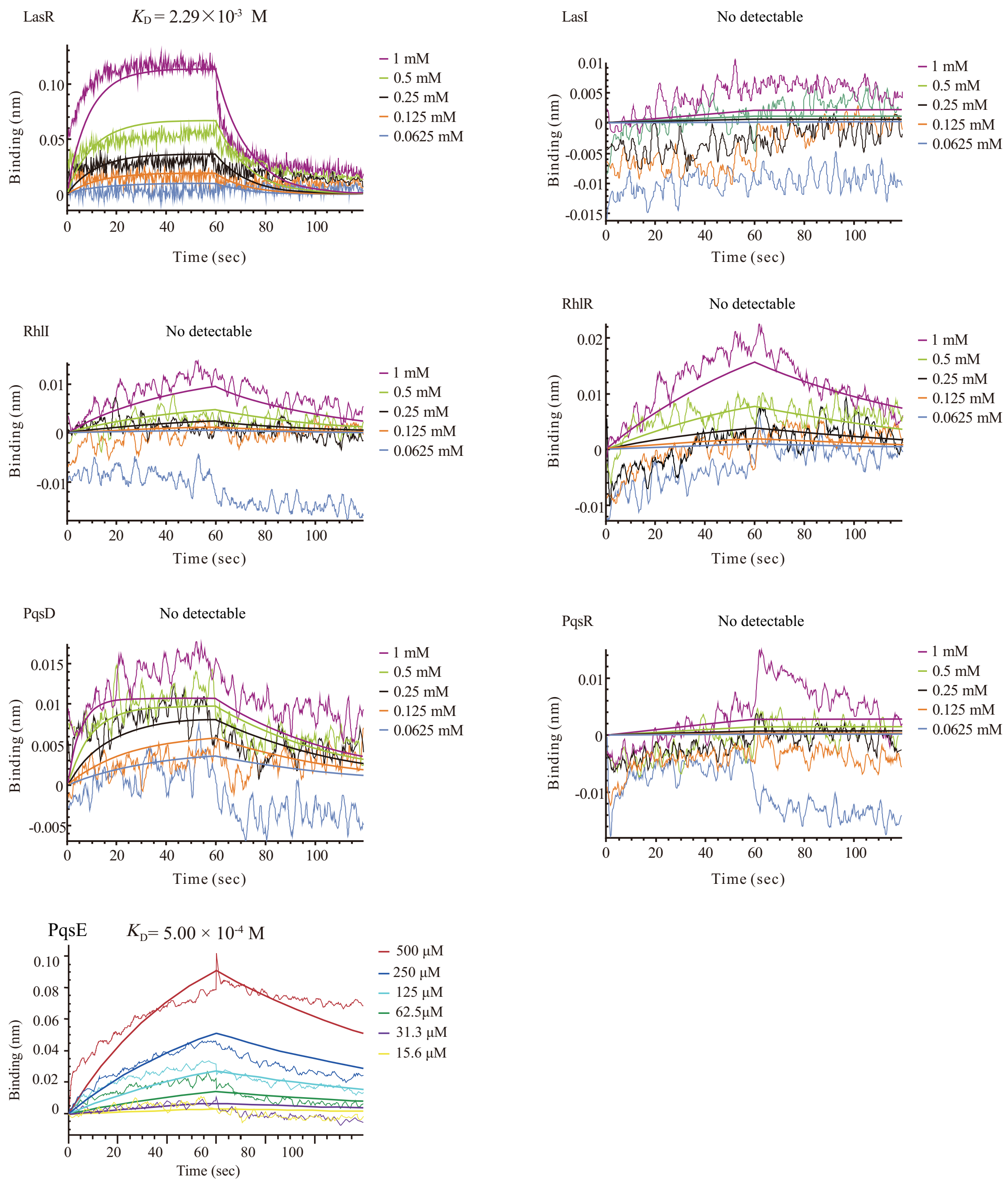

FIG S3 Kinetic binding sensorgrams of norharmane with LasR, LasI, RhlI, RhlR, PqsD, PqsR, and PqsE respectively.

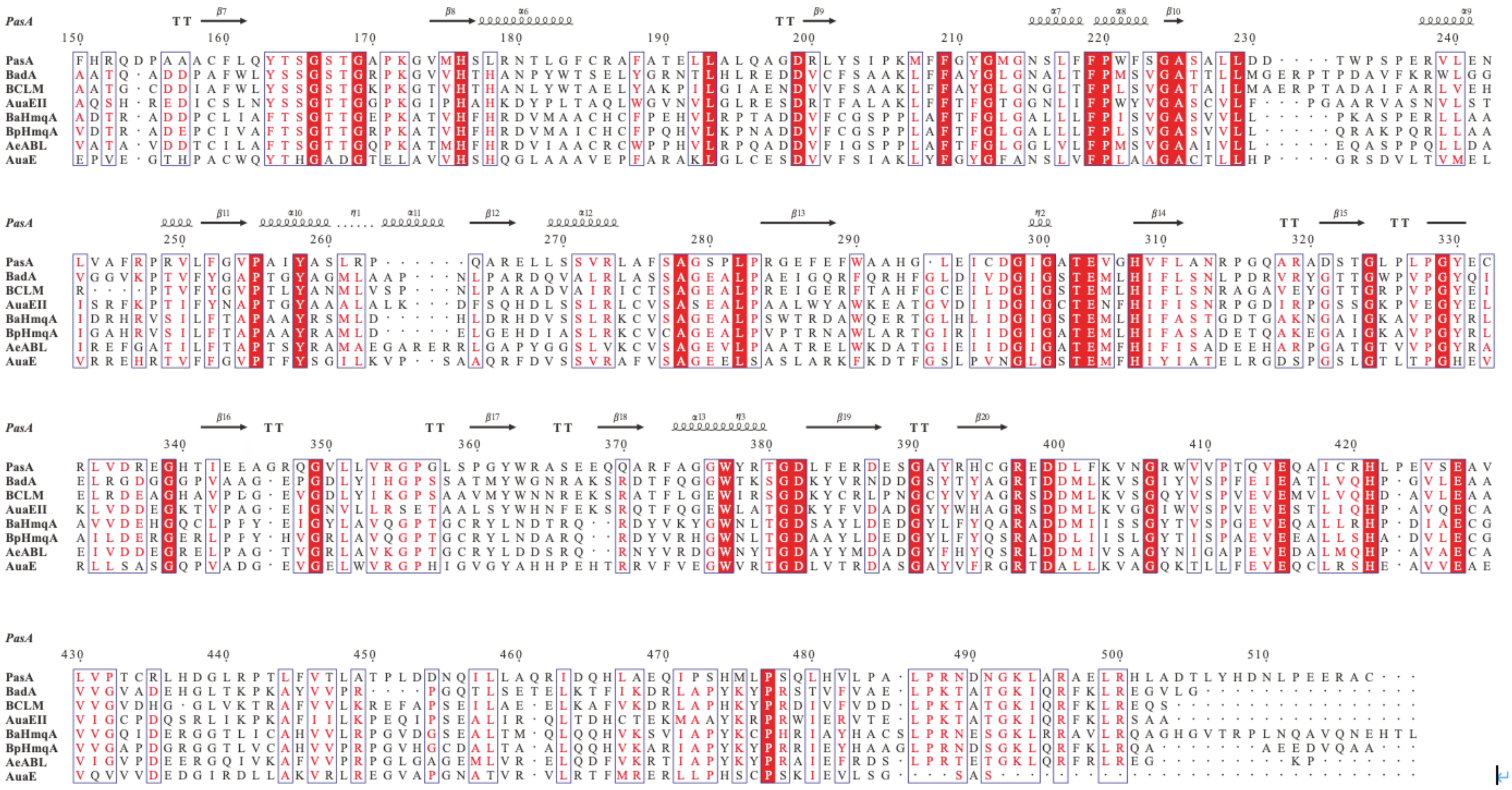

FIG S4 Sequence alignment of PqsA (residues 155-500), anthranilate-CoA ligases (AuaEII, BpHmqA, BaHmqA, AeABL), and acyltransferase (AuaE). Protein sequences were aligned with Clustal X2 and the figure was generated using ESPript3

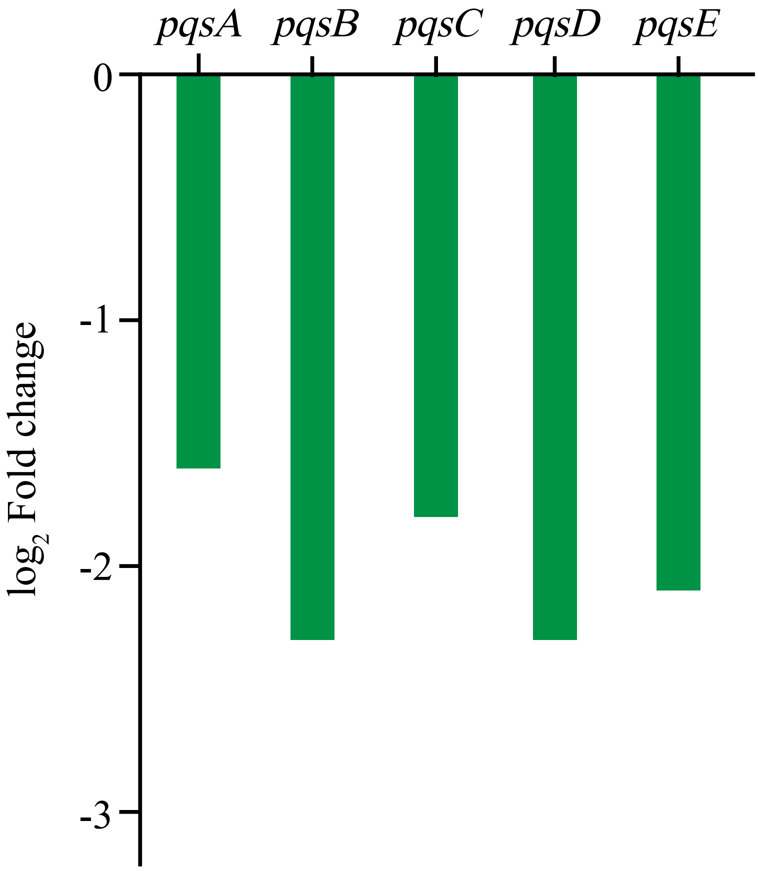

FIG S5 Transcriptome analysis of the genes of norharmane-treated *P. aeruginosa* involving in AQ biosynthetic pathway.

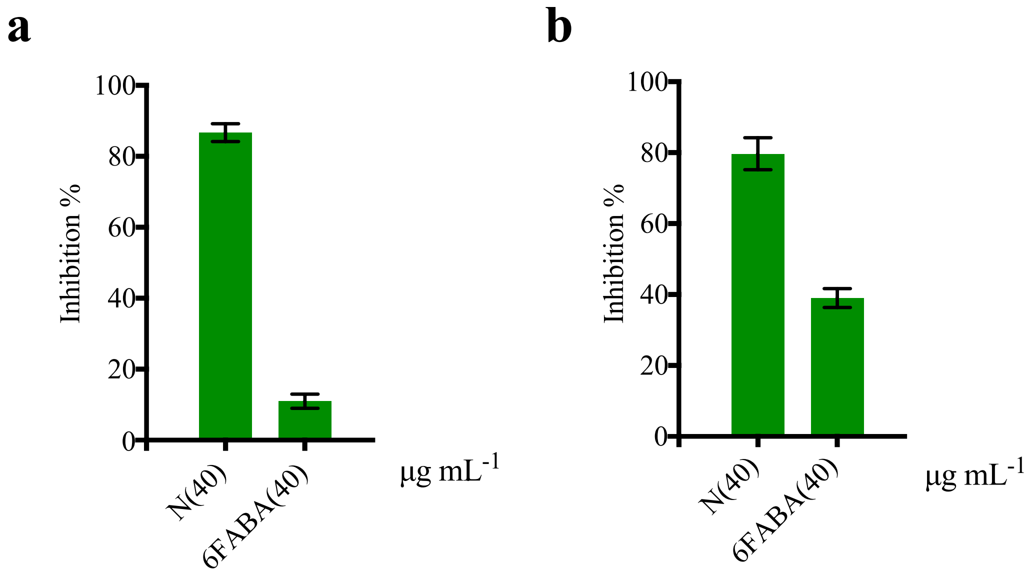

FIG S6 Inhibition of pyocyanin production and biofilm formation in *P. aeruginosa* treated by norharmane (N) and 6FABA. (a) The effect of *P. aeruginosa* treated by norharmane and 6FABA in pyocyanin production. (b) The effect of *P. aeruginosa* treated by norharmane and 6FABA in biofilm formation.

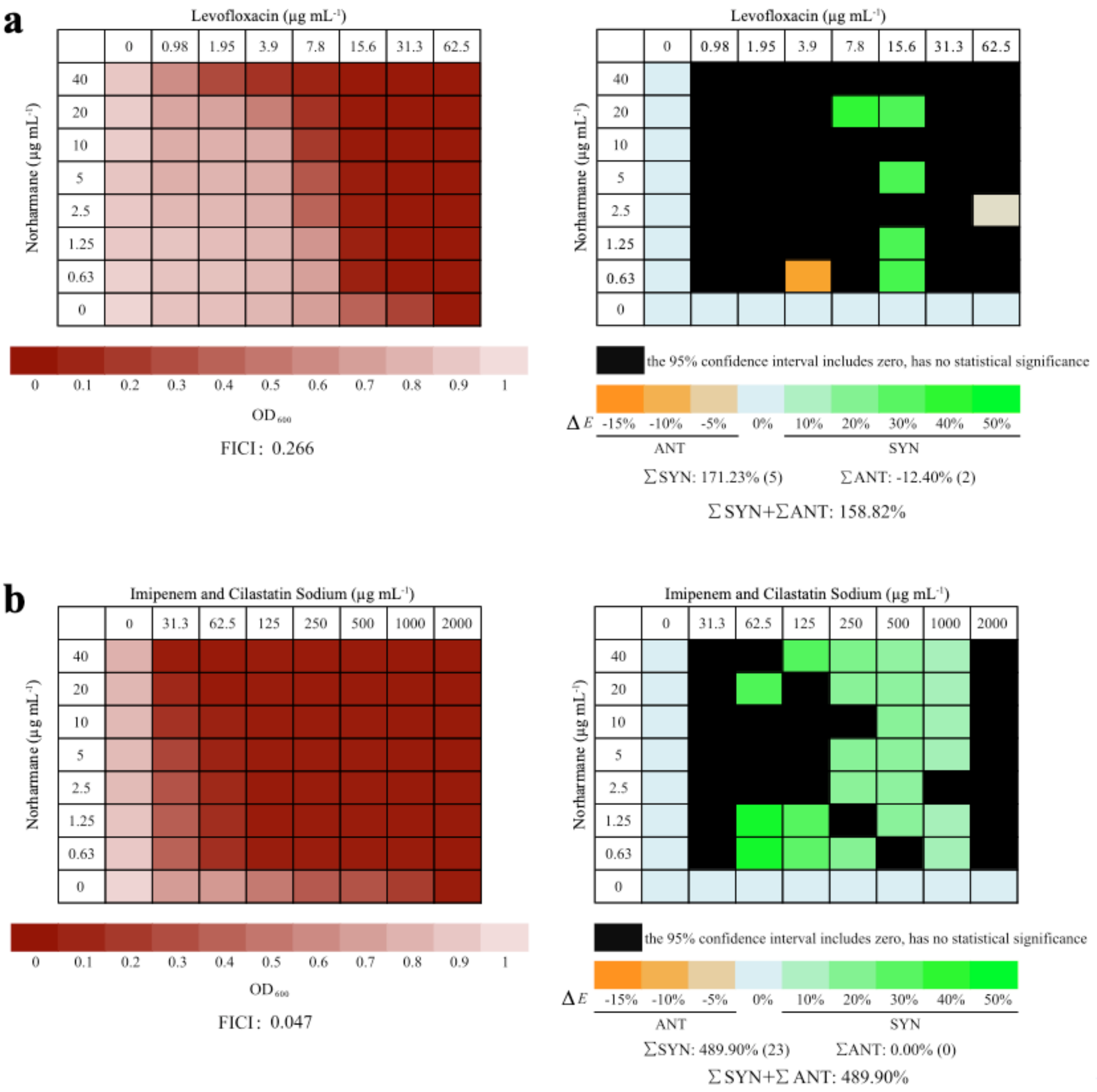

FIG S7 Synergism between norharmane and three antibiotics was evaluated against *P. aeruginosa* C218 by FICI and Δ*E*. (a) Synergism between norharmane and levofloxacin hydrochloride was evaluated against *P. aeruginosa* C218 by FICI and Δ*E*. (b) Synergism between norharmane and imipenem and cilastatin sodium was evaluated against *P. aeruginosa* C218 by FICI and Δ*E*. Experiment were independently conducted three times with similar results.

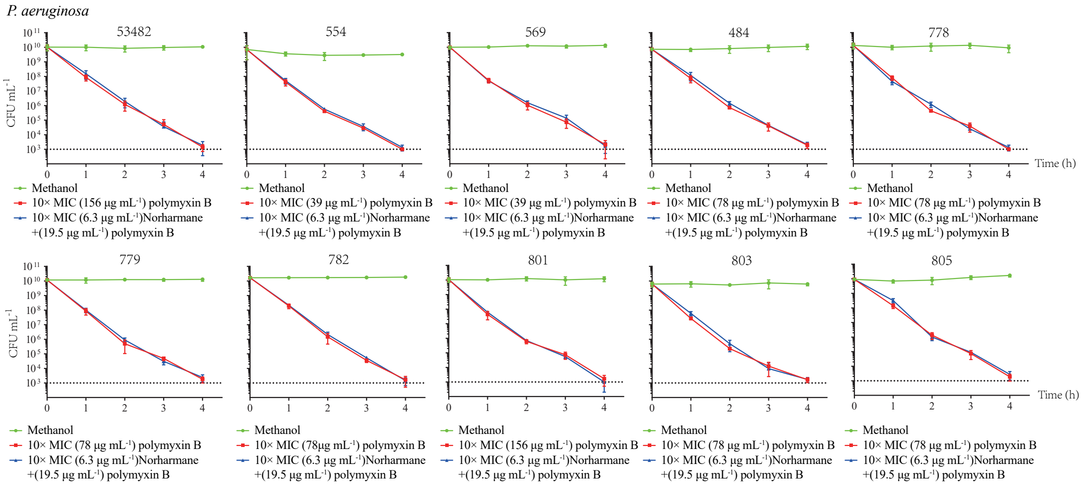

FIG S8 MDR *P. aeruginosa* were treated with polymyxin B and norharmane-polymyxin B. CFU counts of bacteria were calculated by serial dilution and followed by drop plating on LB plates. Individual data points (n=3) and mean±s.d. is shown

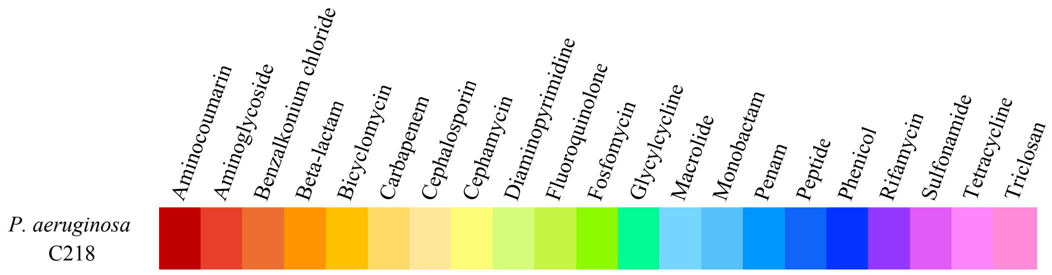

FIG S9 Distribution of ARGs in *P. aeruginosa* C218. The classes of ARGs were detected by bioinformatic tools ResFinder 3.2 (https://cge.cbs.dtu.dk/services/ResFinder), and CARD (https://card.mcmaster.ca/home)

Supplementary Experimental Procedures:

Strain identification of marine-derived *Microbactrium* sp. 40DY182. Marine-derived *Microbactrium* sp. 40DY182 was identified as an *Microbactrium* sp. based on 16S rRNA sequence comparison (GenBank Accession Number MK002745.1). It had been deposited in the Guangdong Microbial Culture Center (GDMCC, China) under restricted access.

Extraction and isolation. A total of 80 L of bacterial cultures were cultivated in a 100 L fermentor for 2 days. The medium supernatants were extracted three times with ethyl acetate. The extract was purified by reversed-phase C_{18} preparative HPLC (30 min linear gradient from 10% to 100% in methanol in water) to yield six fractions. Fraction 5 was further purified by reversed-phase C18 semipreparative HPLC (30 min linear gradient from 30% to 100% methanol in water) to yield five fractions. Fraction 1 was purified by reversed-phase C_{18} semipreparative HPLC (135 min linear gradient from 20% to 100% methanol in water) to yield one pure compound: **1** (15 mg, t_R = 22 min).

Norharmane (1): White solid. ^{1}H NMR (500 MHz, DMSO-d_6) δ 11.62 (s, 1H), 8.92 (d, *J*=0.96 Hz, 1H), 8.34 (d, *J*=5.18, 1H), 8.24 (m, 1H), 8.12 (dd, *J*=5.22, 1.09, 1H), 7.61 (m, *J*=8.19, 2H), 7.24 (m, 1H). ^{13}C NMR (125 MHz, DMSO-d_6) δ 140.52, 138.11, 135.97, 134.02, 128.09, 127.42, 121.78, 120.60, 119.23, 114.63, 111.94. HRESIMS *m/z* 169.0757 $[M+H]^+$ (calcd for $C_{11}H_9N_2$ 169.0767).

Whole genome sequencing of MDR *P. aeruginosa* C218. The puriLink Genomic DNA purification kit (Invitrogen Corp.) was used to purify genomic DNA from LB Agar culture plates of MDR *P. aeruginosa* C218. The harvested DNA was detected by the agarose gel electrophores and quantified by Qubit® 2.0 Fluorometer (Thermo Scientific). Sequencing libraries were generated using NEBNext® UltraTM DNA Library Prep Kit for Illumina (NER, USA) following manufacturer's protocol. PCR samples were purified (AMPure XP system) and libraries were analyzed by Agilent 2100 Bioanalyzer and quantified using real-time PCR. The whole genome of *P. aeruginosa* was sequenced using Illumina NovaSeq sequencing platform.

Raw data obtained had a certain proportion of low quality data. In order to ensure the accuracy and reliability of the subsequent information analysis, the raw data must be filtered to obtain cleandata. Reads with any one of the following characteristics were discarded: (I) adapter contamination (≥ 15bp); (II)Reads with low quality bases (mass value⩽20) over a certain percentage (the default ≥40%); (III) Reads with too many N bases (>10%).

Antibiotic synergy test. The microdilution checkerboard assay was used for determining synergy of norharmane with clinical antibiotics against *P. aeruginosa*. Briefly, serial twofold dilutions of norharmane were combined with serial twofold dilutions of each clinical antibiotic, creating an 8×8 matrix in a 96-well (8×12) microtiter plate. The concentrations of norharmane and antibiotics used were determined based on sub-MIC and MIC, respectively. The calculation of FICI as follows: FICI=Sub-MIC of norharmane in combination / Sub-MIC of norharmane alone + MIC of antibiotic in combination / MIC of antibiotic alone. The interaction between two compounds was defined, as follows: synergy if FICI≤0.5, addition if 0.5 < FICI≤1, no interaction if 1 < FICI≤2, antagonism if FICI

> 2. The interaction between the predicted and the measured percentage of growth at various concentrations (ΔE) was calculated as follows: ΔE= percentage of growth of norharmane alone × percentage of growth of antibiotic alone－percentage of growth of drug combinations. The interaction between two compounds for each well was defined, as follows: statistical synergy when $\Delta E > 0$ as well as its 95% confidence interval among the three replicates was positive, statistical antagonism when $\Delta E < 0$ as well as its 95% confidence interval among the three replicates was negative. Total synergistic (ΣSYN) and antagonistic (ΣANT) interactions were calculated. No interaction if interactions with≤100%, synergy if interactions with > 100%, antagonism if interactions with < -100%. The assay was conducted in triplicate.

Drug resistant detection of MDR *P. aeruginosa* C218. After the determination of MICs of clinical antibiotics against *P. aeruginosa* C218 (Table S2), MDR *P. aeruginosa* C218 will be defined based on Performance Standards for Antimicrobial Susceptibility Testing of Clinical and Laboratory Standards Institute (28th Edition). *P. aeruginosa* C218 is susceptible to tobramycin (MIC 3.9 μg mL^{-1}), and is intermediate susceptible to gentamycin (MIC 9.75 μg mL^{-1}), amikacin (MIC 15.6 μg mL^{-1}), polymyxin B (MIC 7.8 μg mL^{-1}), neltimicin (MIC 31.25 μg mL^{-1}), and colistin (MIC 3.9 μg mL^{-1}). However, *P. aeruginosa* C218 is resistant to 19 antibiotics belonged to six different antimicrobial categories. Therefore, *P. aeruginosa* C218 is defined as MDR *P. aeruginosa*.

Cytotoxicity. Human liver hepatocellular cells, HK-2 (ATCC CRL-2190), human umbilical vein endothelial cells, HUVECs (ATCC PCS-100-013), human liver *cells*, *HL-7702* (Chinese Academy of Sciences Committee Typical Culture Collection Cell Bank, Shanghai, China, Catalog # GNHu6), human hepatoma cells, HepG2 (Chinese Academy of Sciences Committee Typical Culture Collection Cell Bank, Shanghai, China, Catalog # TCHu72), and human lung fibroblast cells, MRC-5 (Chinese Academy of Sciences Committee Typical Culture Collection Cell Bank, Shanghai, China, Catalog # GNHu41) were maintained in Dulbecco's modified eagle medium (DMEM) containing antibiotics penicillin-streptomycin (100 U mL^{-1}, Gibco, CA, USA) and 10% fetal bovine serum, FBS (Gibco, CA, USA), respectively in a humidified 5% CO_2 incubator at 37°C, followed by MTT tests. The absorbance was measured using a Multiskan Spectrum Microplate Spectrophotometer (Bio-Rad, California, USA) at 490 nm. The percent absorbance relative to that of the untreated control was calculated. The assay was conducted in triplicate.

Mouse lung infection model for evaluating drug efficacy and toxicity. A previously described experiment to mimic a mouse lung infection was used with modifications (1). Five-week female CD-1®(ICR) IGS mice (20±2 g) were obtained from Qinglong Mountain Animal Breeding Center (Certificate # SCXK(Su) 2017-0001, Nanjing, Jiangsu, China). Mice were allowed to acclimatize for 1 week. To make mice neutropenic, 150 mg kg^{-1} of cyclophosphamide on day 4 and 100 mg kg^{-1} of cyclophosphamide on day 1 were administered via IP injection before infection, respectively, following 12 h phase shift in the light/dark cycle. *P. aeruginosa* C218 was grown in the LB for 20~24 h (about

6×10^8 CFU mL^{-1}). On the day of infection, each mouse was infected with ~10^7 cells of stationary-phase MDR *P. aeruginosa* C218 by laryngeal trachea under complete anesthesia. At 24 h post-infection, mice received treatment with polymyxin B and norharmane alone or in combination every 12 h for 24 h via IP injection. Control mice were injected with 200 μL of DMSO/Tween 80 in saline every 12 h for 24 h. After euthanizing mice at 36 h post-infection, blood was collected from eyeball and treated with an anticoagulant. Serum was analyzed for glutamic pyruvic transaminase (ALT), and creatinine (Cre) with *Automatic Biochemical Analyzer (DXC-800,* Unicel*, Beckman Coulter,* USA), following the manufacturer's protocol. Henry rate method was used to detect ALT, and picric acid method was used to detect Cre. Lung tissue was homogenized in saline and the number of MDR *P. aeruginosa* C218 in homogenates was enumerated by serial dilution and plating on MH media. The bacterial burden was recorder as CFU mL^{-1}. All experimental procedures were conducted in accordance with the Guide for the Care and Use of Laboratory Animals in the Zhejiang University of Technology, Hangzhou, China, and conformed to the National guidelines. A sample size of mice per group was calculated for a means±standard deviation. *P* values were determined using an unpaired, two-tailed Student's t-test.

References

1. Pantel, L. et al. Odilorhabdins, antibacterial agents that cause miscoding by binding at a new ribosomal site. *Mol. Cell.* **70**, 83-94 (2018).
